# Personal and familial needs of parents with toddlers: a qualitative study exploring the needs of parents in urban Bengaluru, India

**DOI:** 10.3389/frcha.2026.1664652

**Published:** 2026-05-25

**Authors:** Chaithra Holla, Bino Thomas, M. Thomas Kishore, Sphoorthi Prabhu, Manjula Basavaraju

**Affiliations:** 1 Department of Psychiatric Social Work, National Institute of Mental Health and Neurosciences, Bengaluru, India; 2 Department of Clinical Psychology, National Institute of Mental Health and Neurosciences, Bengaluru, India; 3 Department of Psychiatry, Mazumdar Shaw Medical Centre, Narayana Health, Bengaluru, India

**Keywords:** child development, family, India, LAMICs, parenting, personal needs, qualitative study, toddlers

## Abstract

**Background:**

Parenting is multifactorial and existing literature on parenting highlights the role of parental stress, family and environmental factors shaping parenting that influence child development. There is a dearth of literature on the needs of parents with toddlers in the Indian socio-cultural context. Identifying them helps in planning pragmatic interventions.

**Aim:**

The present study aimed to understand the personal and familial needs of parents with toddlers.

**Design:**

The study adopted an exploratory research design using semi-structured interviews. Data was analyzed using thematic analysis.

**Setting and participants:**

The study was conducted in urban Bengaluru, India. Interviews were conducted with eight professionals and ten parents.

**Results:**

The first theme was Personal needs which included sub-themes, namely Lack of personal space, Poor help-seeking behaviour, Emotional experiences, and Coping skills. The second theme was Familial needs, which included sub-themes: Support, Challenges related to communication, Differences in parenting practices, and Grandparenting.

**Conclusion:**

The study findings indicated the need for including the content on parental self-care and family dynamics in parenting programme.

## 1 Introduction

Parenting has received significant attention from researchers across various disciplines. Various theoretical frameworks emphasized the role of parenting in overall child development and motivated many research studies investigating the impact of parenting on child development for over 75 years (1). A range of factors influences parenting behaviour, including parental functioning (2), socioeconomic resources, education, employment, parental health, child characteristics (3, 4), and family environment (5). Kwon et al. (6) studied concerns of parents with toddlers and found that parenting stress stems from toddlers' typical but challenging developmental characteristics such as autonomy, stubbornness, non-compliance, limited language ability and parental specific issues such as time management, and inconsistent parenting.

As parenting is multifactorial, parents expressed challenges while responding to children's emotional and behavioral needs (7, 8) and the lack of preparedness for the transition to parenthood makes parenting all the more complex (9). Studies have also highlighted the impact of parenting young children on personal life (10), quality of the couple's relationship (11, 12), and the mental health of parents (13, 14).

In the traditional Indian family system, although the mother was expected to take on most childcare responsibilities, she was never left alone to provide childcare (15). The parents, and other extended family members shared responsibility of child's upbringing and development (16) which had both benefits and limitations (17). Typically, grandparents, especially the grandmothers, involve in childcare, and this involvement can include material investment, emotional support and promoting family cohesion, as shown in studies from urban Bangalore and other Indian settings (17, 18). In patrilineal and patrilocal systems this may include substantial involvement from paternal grandparents when they live with or near the toddlers.

Many adults or parents do not live in extended families in the changing family systems in India. Thus, there is little opportunity for others from older generations to give advice and emotional support (19). With the changing socioeconomic conditions, women are employed and accepting multiple roles without much paternal involvement in child care (20). Playing multiple roles is naturally stressful for a parent of a toddler. Studies on personal and familial needs of parents with toddlers largely come from the interviews with parents from the high-income countries, highlighting the dearth of research in low- and middle-income countries (LAMICs) especially exploring professionals' perspectives. In this background, the present study attempted to understand the personal and familial needs of parents of toddlers both from professional and parental perspectives.

This study drew on Bronfenbrenner's ecological systems theory (21), viewing parenting influenced by an interaction between individual, family, and socio-cultural contexts, including extended family structures common in Indian households. Within this framework, the challenges described by professionals and parents were understood as indicators of personal and familial needs that need to be addressed to support parents' well-being and stable family functioning.

## 2 Methods

**Figure 1:**
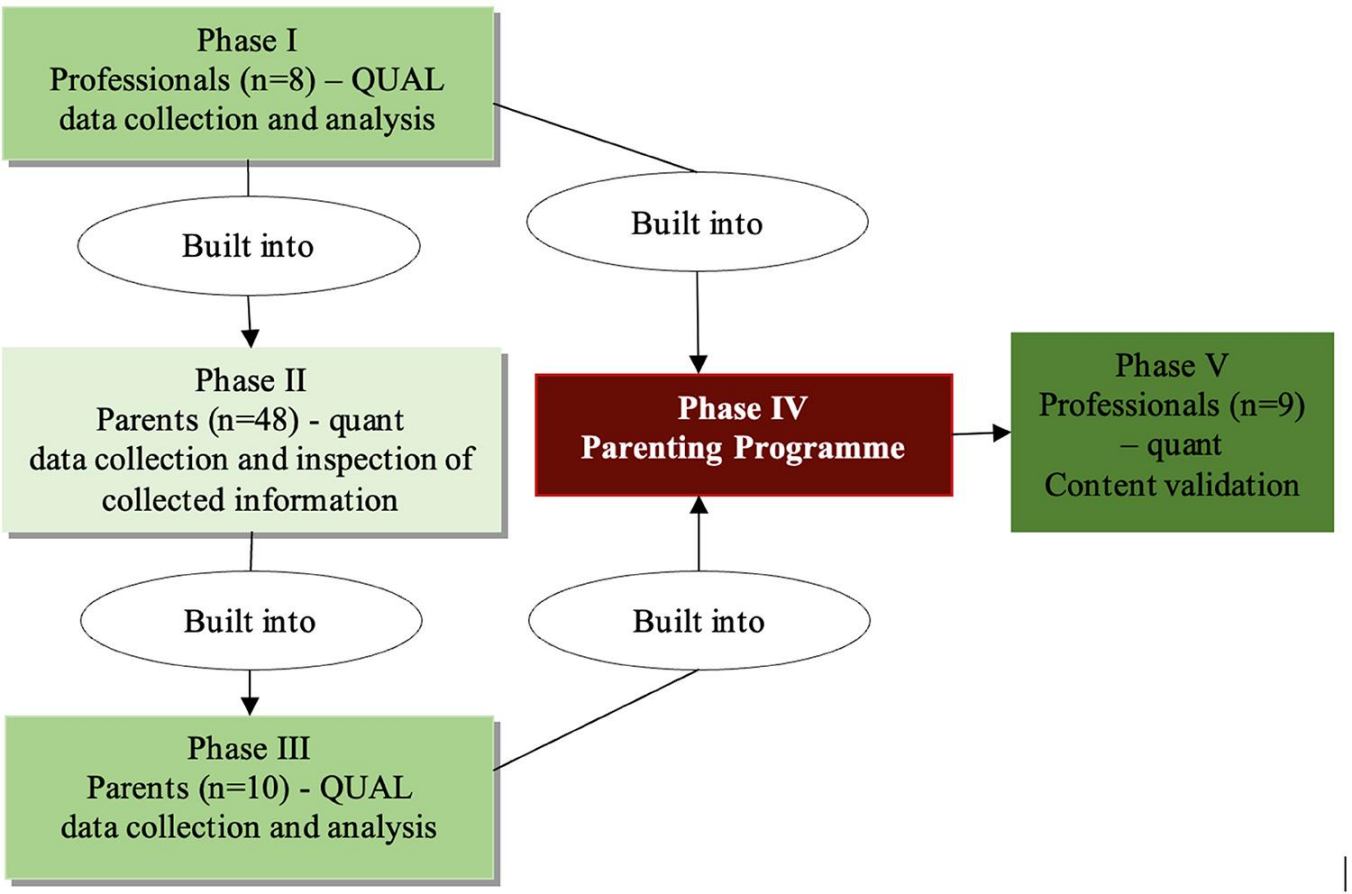
Exploratory sequential design. Figure is original figure by the authors, based on Creswell (47), and the term QUAL (capitals are used for emphasis) denotes a qualitative approach to data collection.

This paper is an outcome of a PhD study of the first author (Figure 1) titled “Development and Content Validation of Parenting Skills Programme for Parents of Toddlers”, and a portion of the qualitative findings has been published elsewhere (8). The study was registered at National Institute of Mental Health and Neuro Sciences (NIMHANS), Bengaluru, and approved by its Human Ethics Committee. The present paper discusses the perceived personal and familial needs of parents of toddlers, as described by professionals and parents in Urban Bengaluru.

### 2.1 Participants

Participants included (1) mental health professionals (*n* = 5), pediatricians (*n* = 2), and ICDS (Integrated Child Development Services)^1^ officers (*n* = 1) with a post graduate degree in child development or allied subjects, with a minimum of 2 years of experience in early childhood development. (2) Parents of toddlers who qualified and consented for interviews (based on Phase II findings explained in the section below) The parents who spoke Kannada (vernacular language) or English were included (*n* = 10) and parents with psychiatric or medical conditions, single parents, and those with toddlers with special needs were excluded.

### 2.2 Data collection

Data collection involved III phases.

Phase I: This is a qualitative phase wherein 14 professionals were contacted by email or phone for the semi-structured interviews; eight professionals agreed to participate in the study. Six in-person interviews were conducted in Bengaluru, with two being online. The interview focus was to reflect the complex understanding surrounding the central issue of “parenting.” These professionals drew on their diverse clinical, and community-based experience with both mothers and fathers when describing parenting challenges.

Phase II: The purpose of this quantitative phase was to select the parents for semi-structured interviews. 48 parents had responded to tools capturing socio-demographic details (details in Supplementary Material), parenting practices, and toddlers' socio-emotional development. After inspecting their responses to these tools, 27 parents were approached to participate in the qualitative interviews (Phase III).

Phase III: In this qualitative phase, semi-structured interviews with parents of toddlers (12–36 months) explored everyday parenting challenges. Interviews were conducted at the toddlers’ preschool (*n* = 8), at home (*n* = 1) and online platform (*n* = 1). Nine interviews involved mothers; only one interview involved both the parents. During parental interviews, the toddlers were present and engaged in play by another attendee (preschool staff or a relative), albeit within the vicinity.

The qualitative data collected at Phase I and III was used for this paper.

### 2.3 Data collection tools

Socio-demographic details were collected at the beginning of the interviews. Semi-structured interview schedules (SSISs) (22) were used with professionals (SSIS-Pr) and parents (SSIS-Pa). The SSIS-Pr and SSIS-Pa contained a set of open-ended questions with prompts under each question and probes. The probes were either summary techniques wherein the researcher summarises what the participant has said to check the correctness of understanding or non-directive probing like “Could you give an example?”, “Could you elaborate on this?”. The researcher asked participants questions as they appeared in the SSIS-Pr and SSIS-Pa. The questions in SSIS-Pr focused on professionals' experiences working with the parents of toddlers and their concerns about parents (fathers and mothers). The questions in SSIS-Pa focused on parents' understanding related to child development, challenges while parenting toddlers, parenting practices, support system.

Both SSIS-Pr and SSIS-Pa were developed based on the literature. For SSIS-Pa, themes that emerged from professional interviews were also considered. SSIS-Pr was face validated by three Psychiatric Social Workers and one Child Psychiatrist. SSIS-Pa was face validated by two Psychiatric Social Workers.

Interviews, lasting 45–60 min, were audio-recorded with their consent, transcribed, and analyzed iteratively to develop codes. The recorded interviews conducted in English were transcribed into English text, interviews that were conducted in Kannada were transcribed into Kannada text and translated into English text, and field notes were added to this. Each interview was analysed to extract additional codes before moving to the next interview and added to SSIS-Pr and SSIS-Pa to ask the next participant. Data collection continued until saturation (23). Interviews were conducted from June 2019 to March 2020. Permission from the preschools and written informed consent forms were obtained from all the participants.

### 2.4 Data analysis

Thematic analysis with a 15-item checklist by Braun and Clarke's (24) was followed and the analysis was conducted manually. The data analysis began with familiarizing data, followed by creating codes. The codes that were similar in conceptual meaning were combined and used with the rest of the transcripts. Codes were categorized into subthemes and themes (Table 1) and reviewed by two peers for consistency (24, 25).

**Table 1:** Themes and subthemes.

Themes	Subthemes
Personal needs	Lack of personal space
	Poor help-seeking behaviour
	Emotional experiences
	Coping skills
Familial needs	Support
	Challenges related to communication
	Inconsistency in parenting
	Grandparenting

## 3 Results

### 3.1 Socio-demographic profile of the participants—professionals and parents of toddlers

The mean (SD) age of the professionals (*n* = 8) was 37.71 (±7.16), and had an experience of 12.87 (±9.68) years in the field of child development and/or parent education and support. Five held a doctoral degree in psychology or social work or nursing while the rest had a DM (Doctorate of Medicine) in Child and Adolescent Psychiatry. Parents (*n* = 10) with a mean (SD) age of 29.5 (±3.66), had a monthly family income of INR 148,000 (±78,852), most held a bachelor's degree or above (9/10), many were homemakers (7/10) and lived in extended families (7/10).

The themes and sub themes that emerged from the consolidated data of both professionals and parents are presented below. While we had ten parent participants who spoke about their experiences, professionals shared their perspectives from their rich experience of working with parent population of diverse backgrounds. In most responses, professionals referred to both mothers and fathers while explaining their perspectives which are indicated as “parents”.

### 3.2 Personal needs

The theme portrays the effects constant demands of parenting toddlers can have on parents' personal needs. The parents experience guilt and anxiety and require support to cope with these emotions.

#### 3.2.1 Lack of personal space

Professionals reported that the demanding developmental needs of toddlers make parents spend most of their time in toddlers' care. Additionally, one professional said, the parents carry over their job fatiguability (referring to working parents, might be mothers or fathers) to home and require some personal space to relieve their stress. The professional described parents' multiple responsibilities as “torn between bringing up the toddler and other domestic responsibility.” Some parents take “Me time” while the toddlers are engaged in playgroup or playdate; however, the social expectation for the mothers to take most of the childcare responsibilities and lack of paternal involvement make them feel overwhelmed, as reported by both the professionals and the parents. Although some mothers, as reported, are now more confident leaving toddlers with fathers than they were during infancy, they never felt relieved of childcare.

“Take a break; there is nothing wrong to take a break from children. Like, 1 or 2 days leave them with grandparents and take their [parents] time off or maybe even for a few hours.” Professional 2

“Even I want free time. My husband prefers to do any other work at home rather than looking after him [toddler]. He cannot handle it when the child cries. I agree that I understand my child better than him [husband], but it is frustrating to be with the child all the time.” Mother048

A professional explained how some parents withdraw from social activities, which gradually may increase feelings of isolation, in the early years of parenting.

#### 3.2.2 Poor help-seeking behaviour

Most professionals said that parents do not seek enough help from their families or professionals. They try to do a lot of work by themselves, often overdo things for their toddlers instead of training the child in age-appropriate activities. For example, parents may continue to feed and clean toddlers instead of gradually teaching these self-care skills, in order to avoid spills and frustration. They may also discourage messy play for similar reasons and prefer to keep toddlers constantly engaged themselves. These increase parental stress adding to the existing problems such as lack of personal space.

“Unfortunately, I see more denial than help-seeking in today's generation. Help-seeking itself is stigmatized in India.” Professional3

While professionals described this pattern as poor help-seeking, a working mother expressed it as struggling to balance multiple roles and its impact on her, describing how routine household activities, child care and professional duties contributed to poor concentration. Further, she said she was unable to be fully mindful of either her household chores or the time spent with her toddler.

“It is difficult to handle both work and household chores and feeding, cleaning her [toddler], getting her [toddler] ready for school, and then going to work. It will be a crisis at home if she [toddler] falls sick. This juggling so many things affect my concentration at the office.” Mother003

Another mother said she shared a deep emotional attachment with her child, to the extent that she felt to meet all of her toddler's needs by herself. She noted that this sense of obligation made her feel overwhelmed and fatigued while managing various responsibilities. In both instances, the mothers could have sought help from their families but chose not to, which indicates poor help-seeking behaviour among mothers.

#### 3.2.3 Emotional experiences

Parents echoed the professionals' observation that parents, including stay-at-home mothers, often feel guilty for not giving full attention to their toddlers. The guilt stems from the belief that parenting requires their undivided time, energy, and constant patience.

“Parents feel they need to be with the child all the time and pay complete attention, which is practically not possible. The parents may be preoccupied with something, and later they feel guilty about it.” Professional8

“But when I have to completely dedicate myself to something for her (toddler)—like doing something with her from morning to evening someday, taking her out for an outing, or sitting with her to teach her something—and I wouldn't have time for it, I feel bad the whole day for not doing it.” Mother003

Parents said they sometimes respond to their toddlers based on their mood, which might be influenced by work stress, illness, or personal conflicts and feel guilty after reacting harshly to their child.

“Sometimes, I feel angry at him [toddler] because I will also not be in a good mood all the time. It may be because of mood swings during menstrual periods. During such times I find it difficult. I worry about getting angry with him [toddler]. Later I feel angry at myself and guilty.” Mother048

Many parents avoid allowing children to experience age-appropriate exploration during play, peer activities, fearing its negative effects. Safety concerns also contribute to parental anxiety which often leads to overprotectiveness or restrictive environments.

“I feel worried after getting to know about certain incidents that happened with girls. I often worry what if the school gate is open, and what if someone takes her [toddler] away? I do not let her [toddler] go to anybody's house in our building.” Mother019

One professional said parental anxiety often acts as a barrier to seeking professional help for socio-emotional developmental delays. In India, one of the pathways to child psychiatry is referral from a paediatrician, and paediatricians may sometimes hesitate to refer a child because of parental anxiety about their child receiving a psychiatric label. The professional emphasised that a temporary period of heightened parental anxiety is manageable compared with delaying referral, which can worsen the child's socio-emotional or neurodevelopmental difficulties.

“I ask pediatricians ‘why have not you [paediatrician] referred early’, they say ‘the parents were very anxious.’ I feel that the anxiety of 2 months is better than the long-term damage you will do.” Professional5

Parental anxiety about the child's wellbeing and development results in their imposing rigid practices with the child. The professional recalled an instance wherein the parents constantly insisted on their child to wash hands before doing any activity or repeated instruction not to touch things.

“So, I said, ‘why so much.’ They said, ‘ma’am, but he can fall ill.’ I examined the parents; the parents were anxious” Professional5

Another mother expressed insecurity if her toddler becomes more attached to the grandparents, particularly given her strained relationship with her in-laws.

“I do not like if she [toddler] prefers someone else over me. She [toddler] has to be with my in-laws when I start my career and I feel insecure sometimes; what if she [toddler] starts to love them [in-laws] more than me.” Mother034

#### 3.2.4 Coping skills

The professionals expressed that poor coping skills often lead to irritability and aggression among parents, which teaches toddlers to use aggression for problem-solving. Two professionals said, parents' perceived self-efficacy, competency, and self-esteem are important while rearing young children.

“How to cope as a parent, stress management skills, competency also can be a part of how well they can parent.” Professional6

### 3.3 Familial needs

The joint or extended family systems in the Indian households presents both advantages and limitations for the parents. The theme describes how family dynamics such as communication among family members, differences in parenting, parental insecurities affect parenting. On one hand, extended families offer support to toddler care, including informational support and show warmth and affection. On the other hand, intergenerational differences in child-rearing practices and poor communication can result in other family members withdrawing from caregiving. Differences of opinion, lack of consensus, altercations between the father and mother strain the couple's relationship and negatively influence their parenting.

#### 3.3.1 Support

The extended family system supports parents, especially working mothers, ensuring child safety. The professionals suggested that maintaining harmonious relationships among the family members, help balance responsibilities, and toddler care. Working parents can be at greater peace of mind at the workplace when their toddlers are cared for by extended family members rather than by hired nannies.

“I feel grandparents are the safest option for childcare for one and a half years. You hire a nanny; at least there is someone to supervise.” Professional5

However, professionals highlighted that not all parents in extended families receive support. For instance, one mother reported a lack of help from her in-laws, explaining that experiences can vary even within a similar family structure.

“Although we are staying together, I am not getting enough help from anyone. My in-laws are busy with their lives and activities.” Mother003

Professionals agreed that fathers' involvement in childcare is increasing, though opinions varied on their level of involvement. Some stay-at-home mothers observed that fathers were engaged in play and household chores, while others reported a lack of such involvement. Both the professionals and parents also emphasised the absence of paternal involvement in childcare in certain families.

“When I dig into some details then it becomes obvious in certain cases that one of the parents is not being involved. Majority of the times yes, the noninvolved parent is the father. Very occasionally it has been the mother, when either she has had some psychiatric issues herself, that's the only scenario but father yes, either they are busy with work etc.” Professional7

“My husband does not speak much. He [husband] does not have much time. So, he [husband] plays on Sundays with him [toddler]. He [husband] will be available only for 10 min on weekdays when my child [toddler] returns from school [nursery/preschool]. He [husband] does not help me in anything such as putting the child [toddler] to sleep, feeding.” Mother006

In this study, employed mothers often took on most childcare and household duties, despite a certain amount of involvement from their partners such as sharing parenting information in some families.

#### 3.3.2 Challenges related to communication

Some professionals observed that fathers and mothers often blame each other for their toddler's behavioural issues, with arguments arising even over simple topics like feeding or sleep routines. One professional highlighted that children from abusive families may internalize conflict as a problem-solving strategy, potentially repeating it with peers or partners in adulthood.

“When the parents share a discordant relationship, it obviously has an impact on children. Most of the time, the parents are also caught up in their problems, and their children do not get the required attention.” Professional8

The professionals said that both mother and father need to provide a stable, nurturing environment that makes toddlers feel secure. Professionals advised parents to avoid conflicting discipline and arguments in front of children, instead resolving disagreements privately. One professional pointed out that limited communication between parents due to time constraints, shift work, or a lack of familiarity in interacting as a couple can weaken their relationship and contribute to conflict.

“I think very importantly, it has to be a happy nurturing environment. Children [toddlers] behave the best when they feel the best, endure the best when they know they are loved by both [parents]. Children who grow in difficult and hurting families, you know a child tends to feel guilty for problems – ‘is it because of me [toddler] that my parents are fighting’ - and then children tend to grow with those feelings of guilt and feelings of inadequacy when they have parents who are spotting or fighting all the time.” Professional4

A mother mentioned that her spouse would sometimes blame her for mistakes related to the toddler's care. Professionals emphasized that effective, respectful communication between parents is essential for a supportive, stable environment that fosters healthy child development. Some parents sometimes noticed change in their relationship after their toddler's birth.

“We used to speak about many things before his [toddler] birth, but now it is limited to his [toddler] topic. I also feel that we argue more now on issues concerning him [toddler].” Mother009

One mother opined that poor family communication can lead to grandparents withdrawing support or inconsistent parenting. Some mothers reported to experience tensions due to intense arguments with in-laws, disagreements over decisions whereas some wait until the child is asleep to discuss parenting decisions together with their partner.

“I made restrictions by not letting them [in-laws] show mobile or give chocolates. I used to tell it a bit directly, I think, which made them [in-laws] feel that I was dominating. They [in-laws] were like, ok, it is her child and let her look after the child.” Mother003

#### 3.3.3 Differences in parenting practices

Many professionals noticed differences in parenting between parents and grandparents. Grandparents are often lenient with toddlers, which sometimes conflicted with the parents' approach. In families where both parents are employed, toddlers tend to spend more time with grandparents, making it harder for parents to ensure the child's compliance with their requests.

Some parents struggled to establish sleep and feeding routines, as in-laws preferred accommodating the child's desires rather than enforcing structure. For instance, a mother shared that her in-laws opposed her efforts to encourage self-feeding, preferring instead to distract the child to make feeding easier.

“Another situation is when they playfully say, ‘I will smack you,’ I do not like that because that is how the children learn to smack. At my home, my in-laws and my practices do not match most of the time.” Mother048

Similar concerns were raised about differences in parenting between parents as well. Conflicts often arise when each parent follows a different parenting style. Typically, fathers spend time with toddlers in the evening and tend to be more permissive, while mothers are stricter. Professionals emphasized the importance of parents presenting a united front in their approach to parenting toddlers.

“If one parent is very liberal, one parent is autocratic, and it does make a difference in the parenting. So, there is a major disagreement resulting in conflicts subsequently affecting the child.” Professional2

Many mothers reported that grandparents, and sometimes fathers, tended to “pamper” the child, that led toddlers to utilize these differences, knowing whom to approach to get their way.

“If I shout at my daughter, she gets on to her [toddler] grandmom or granddad. Now she [toddler] has well understood that if she [toddler] cries, they [grandparents] will ultimately give her [toddler] what she [toddler] wants.” Mother022

However, some parents mentioned they discussed these differences when the toddler was asleep. Professionals discussed the need for the parents to set boundaries with other family members in a non-offending manner.

The professionals acknowledged positives in traditional parenting, through grandparenting, such as allowing toddlers to play messily, play with other children, spill over food and ignoring their harmless behaviour. However, they also pointed out that some rigid practices lacked scientific rationale, like the insistence on applying “Drishti bottu” (a small black dot to ward off bad luck) or giving hot water baths even during summer or the belief that children should not be allowed to cry.

“The previous generation would know ways of interaction, socialization, culture, values and beliefs of a particular place that help the parents develop cultural identity and socialize.” Professional1

#### 3.3.4 Grandparenting

While extended family members often offered support, maintaining boundaries with grandparents was challenging. Many grandparents viewed parenting as a “natural instinct” and found it amusing when parents attended parenting sessions or sought guidance, seeing these approaches as overly rigid or idealistic.

“I had to inform them [grandparents] that I was going to talk about parenting issues with you [interviewer]. They [grandparents] asked me sarcastically, ‘do you need a class to raise your child?’ Mother014

As a result, they sometimes intervened, especially during toddlers' tantrums, which could disrupt parental discipline efforts. One mother felt helpless when her child became frustrated by the grandmother playfully withholding a toy beyond the child's reach, and addressing it felt like “talking back.”

## 4 Discussion

Parenting is shaped by multiple factors, yet research beyond the first postpartum year is limited in India. This qualitative study explored the everyday parenting challenges of parents with toddlers through professionals' and parents' perspectives. These challenges were interpreted as “needs” because they implied parents needing help to manage stress, maintain supportive couple and family relationships, and provide collaborative caregiving within their ecological context.

The parent participants belonged to middle-income strata indicated by their education level, monthly household income, and ability to afford preschool for their toddlers the parents reported several needs within their socio-economic context which are discussed here.

### 4.1 Need for personal space

One of the parental needs identified was to have personal space to relieve their daily stressors, consistent with other study findings (7, 26). The working parents had additional challenges that caused fatigue, poor concentration, and mood fluctuations, affecting their parenting practices, that concurred with other studies (6, 9, 10, 14, 27) In case of mothers, a study has also reported that one of the predictors of maternal fatiguability was low self-care and high parental fatiguability would impact parent-child interaction (2, 28). Eventually, these experiences make the mothers feel alone and disconnected from their social circle in the first two years of parenthood (29).

### 4.2 Need for learning coping strategies

The current study identified the need for handling parental guilt, mood changes, and anxiety to reduce their impact on parenting practices implying the need for effective coping strategies. This becomes important for child development. For example, studies have established a relationship between parental emotional regulation and caring behaviour, and its impact on child development (30–33). This understanding helps support parents with their self-care and coping skills, which further fosters healthy parenting practices.

### 4.3 Need for shared parenting

Like mothers, fathers play an essential role in toddlers' wellbeing (34, 35). The professionals and mothers in this study acknowledged the need for paternal involvement in child-rearing; however, they noted that it was inconsistent and largely limited to playing with the toddlers. Devi and Priya (36) noted that although traditional ideologies persist, fathers were involved in nurturing children's academic and social skills. However, this has less implication to toddlers as academics are less practised with toddler group in India. A qualitative study identified the theme of fathers as “playtime partners” (37) similar to the current study findings. Although there is a change in the concept of fatherhood, gender-based roles in childcare still exist. These findings highlight the need for shared parenting responsibilities, which also creates personal space, specially for the mothers.

The parenting stress and disagreements between spouses can result in changed couple interaction, consistent with findings from various studies (6, 7, 9, 26, 27). The western studies have documented that marital quality fosters positive co-parenting, drawing positive parent-child relationships (38, 39). It is plausible that similar processes operate in Indian families, and our qualitative findings suggest that concordant, low-conflict couple relationships support more consistent, collaborative parenting in this context. Therefore, it is essential to strengthen couple relationships as part of supporting effective parenting.

### 4.4 Need for collaborating with extended family members

As far as extended family is concerned, there are mixed outcomes. Extended families often provided vital support for toddler safety and care like warmth, and affection. Sharma and Kanani (17) noted that extended family members could serve as transitional figures during early childhood or remain as enduring attachment figures, offering stability and support. Grandparents, in particular, played an affectionate role in child-rearing, contributing positively to the well-being of the toddler-parent dyad as reported in the studies by Sudo et al. (40). This aligns with the perspective shared by professionals in the current study especially for working mothers wherein the parents can rely on grandparents for toddlers' safety or to take time off from toddlers or grandparents allowing toddlers for free play or ignoring toddlers' harmless behaviour.

The involvement of extended family was also associated with challenges. Intergenerational differences in child-rearing practices and poor communication led to the withdrawal of family members from childcare responsibilities, creating additional stress for mothers that in turn impacting the child rearing by the mother, her health and maintaining the vicious cycle.

The unique culturally relevant familial need, identified in the study, is to maintain equilibrium within the extended family so that parents and toddlers can benefit from its advantages. More than half of the participants lived in extended family setups, offering opportunities for shared caregiving. A study conducted in Urban Bangalore reported grandparental involvement in childcare, and their support through guidance, emotional support, and participation in family and community activities, highlighting the extended family's role in childcare (18). However, the limitations, such as differences in parenting between two generations, could predict adverse child outcomes (41, 42) and parenting styles affect child development in a similar way across cultures (43). To maximize the benefits of extended family support, it is crucial to collaborate with grandparents and other family members by acknowledging their knowledge while gently introducing contemporary parenting practices in a respectful manner, as suggested by Tan et al. (44).

### 4.5 Need for adapting western parenting practices

With exposure to western parenting practices (45), the mothers in Urban India are aware of the need for self-care. However, on the downside, with this exposure where attachment is understood in the context of the nuclear family system, the mothers in India get uncomfortable when the child is attached to extended family members. It should be noted that a study found a toddler preferring the mother even if she was the secondary caregiver (46). Mothers need to see polyadic interaction where multiple caregivers are attached to the child and its benefits on toddler development.

Bronfenbrenner's ecological systems theory (21) was developed largely in Western contexts, where the nuclear family has often been taken as a reference. The present study is situated in urban middle-class Indian families, and many of the parents live in extended family households. The findings suggest that, while the core ecological concept remains the same, experiences of support and stress are shaped by native family structures. Shared caregiving with grandparents, intergenerational differences in childcare, and the need to maintain equilibrium within the extended family imply the need to interpret Western theories in light of the Indian socio-cultural context.

### 4.6 Strength and limitation of the study

The qualitative nature of the study design limits the generalisability of its findings. The majority of parents in this study were middle-class, urban families living in extended or joint households, and therefore their childcare arrangements may not be representative of the Indian population, particularly parents from lower or upper socioeconomic status, rural backgrounds, or in nuclear families. Many were mothers, and were homemakers, which is not typical of the urban Bengaluru women/mothers, although it is common for mothers to take career breaks to care for their young children; this may limit the generalisability of the findings to employed mothers with older children. Recruitment was influenced by the fact that the participants were parents who enrolled their toddlers in preschools. The challenge of the field visits was to find a common time to interview the fathers. The father who participated in the joint interview did not provide much information, making it challenging to get fathers’ perspectives, about their involvement in specific childcare activities.

Despite these limitations, the study provides detailed description of the needs unique to the cultural context of urban middle class families. This study provides a perspective on parenting in the individual and familial contexts, that has been less explored in India. The findings can be used to build a robust quantitative study, focusing on parental self-care, coping, social support, and couple relationships and their impact on child development.

## 5 Conclusion

Parental self-care and family dynamics play important roles in parenting practices, emphasizing the need for parents to have personal space to alleviate distress and maintain equilibrium in the extended family. Robust evidence needs to be established on the predictors of parenting practices of parents with toddlers and their impact on toddlers' outcomes in India.

## Data Availability

The original contributions presented in the study are included in the article/Supplementary Material, further inquiries can be directed to the corresponding author.

## Footnotes

^1^a major Government of India flagship program launched in 1975 to improve the nutritional, health, and education status of children under six, pregnant women, and lactating mothers.

## References

[1] Kuppens S Ceulemans E. Parenting styles: a closer look at a well-known concept. J Child Fam Stud. (2019) 28:168–81. 10.1007/s10826-018-1242-x 30679898 PMC6323136

[2] Cooklin AR Giallo R Rose N. Parental fatigue and parenting practices during early childhood: an Australian community survey. Child Care Health Dev. (2012) 38:654–64. 10.1111/j.1365-2214.2011.01333.x 22017576

[3] Smith CL. Multiple determinants of parenting: predicting individual differences in maternal parenting behavior with toddlers. Parenting. (2010) 10:1–17. 10.1080/15295190903014588

[4] Waylen A Stewart-Brown S. Factors influencing parenting in early childhood: a prospective longitudinal study focusing on change. Child Care Health Dev. (2010) 36:198–207. 10.1111/j.1365-2214.2009.01037.x 20015278

[5] Rueger SY Katz RL Risser HJ Lovejoy MC. Relations between parental affect and parenting behaviors: a meta-analytic review. Parenting. (2011) 11:1–33. 10.1080/15295192.2011.539503

[6] Kwon K-A Han S Jeon H-J Bingham GE. Mothers’ and fathers’ parenting challenges, strategies, and resources in toddlerhood. Early Child Dev Care. (2013) 183:415–29. 10.1080/03004430.2012.711591

[7] Bloomfield L Kendall S Applin L Dearnley K Edwards L Hinshelwood L A qualitative study exploring the experiences and views of mothers, health visitors and family support centre workers on the challenges and difficulties of parenting. Health Soc Care Community. (2005) 13:46–55. 10.1111/j.1365-2524.2005.00527.x 15717906

[8] Holla C Thomas B Kishore MT. Parenting toddlers: evidences of parental needs from south India. Int J Soc Psychiatry. (2023) 69:2079–86. 10.1177/00207640231188032 37462317

[9] Deave T Johnson D Ingram J. Transition to parenthood: the needs of parents in pregnancy and early parenthood. BMC Pregnancy Childbirth. (2008) 8:30. 10.1186/1471-2393-8-30 18664251 PMC2519055

[10] Jevitt CM Groer MW Crist NF Gonzalez L Wagner VD. Postpartum stressors: a content analysis. Issues Ment Health Nurs. (2012) 33:309–18. 10.3109/01612840.2011.653658 22545638

[11] Bäckström C Kåreholt I Thorstensson S Golsäter M Mårtensson LB. Quality of couple relationship among first-time mothers and partners, during pregnancy and the first six months of parenthood. Sex Reprod Healthc. (2018) 17:56–64. 10.1016/j.srhc.2018.07.001 30193721

[12] Hildingsson I Thomas J. Parental stress in mothers and fathers one year after birth. J Reprod Infant Psychol. (2014) 32:41–56. 10.1080/02646838.2013.840882

[13] Corkin MT Peterson ER Andrejic N Waldie KE Reese E Morton SMB. Predictors of mothers’ self-identified challenges in parenting infants: insights from a large, nationally diverse cohort. J Child Fam Stud. (2018) 27:653–70. 10.1007/s10826-017-0903-5

[14] Gazmararian J Dalmida S Merino Y Blake S Thompson W Gaydos L. What new mothers need to know: perspectives from women and providers in Georgia. Matern Child Health J. (2013) 18:839–51. 10.1007/s10995-013-1308-8 23843170

[15] Seymour SC. Child care in India: an examination of the “household size/infant indulgence” hypothesis. Cross Cult Res. (2001) 35:3–22. 10.1177/106939710103500101

[16] Palriwala R Neetha N. Stratified familialism: the care regime in India through the lens of childcare: stratified familialism and the care regime in India. Dev Change. (2011) 42:1049–78. 10.1111/j.1467-7660.2011.01717.x 22165159

[17] Sharma M Kanani S. Grandmothers’ influence on child care. Indian J Pediatr. (2006) 73:295–8. 10.1007/BF02825822 16816489

[18] Gray PB Longkumer W Panda S Rangaswamy M. Grandparenting in urban Bangalore, India: support and involvement from the standpoint of young adult university students. Sage Open. (2019) 9:2158244019871070. 10.1177/2158244019871070

[19] Thomas S Vijayakumar C Siva R Isaac R. Parenting children under three years of age in a south Indian setting. Pediatr Nurs. (2007) 33:421–6. 18041330

[20] Suppal P Roopnarine JL. Paternal involvement in child care as a function of maternal employment in nuclear and extended families in India. Sex Roles. (1999) 40:731–44. 10.1023/A:1018808718351 12296064

[21] Guy-Evans O. Bronfenbrenner’s ecological systems theory (2024).

[22] Alston M Bowles W. Research for Social Workers: An Introduction to Methods. 3rd ed. London: Routledge (2019).

[23] Fusch PI Ness LR. Are we there yet? Data saturation in qualitative research (2015).

[24] Braun V Clarke V. Using thematic analysis in psychology. Qual Res Psychol. (2006) 3:77–101. 10.1191/1478088706qp063oa

[25] Shenton AK. Strategies for ensuring trustworthiness in qualitative research projects. Educ Inf. (2004) 22:63–75. 10.3233/EFI-2004-22201

[26] Slomian J Emonts P Vigneron L Acconcia A Glowacz F Reginster JY Identifying maternal needs following childbirth: a qualitative study among mothers, fathers and professionals. BMC Pregnancy Childbirth. (2017) 17:213. 10.1186/s12884-017-1398-1 28673272 PMC5496411

[27] Javadifar N Majlesi F Nikbakht A Nedjat S Montazeri A. Journey to motherhood in the first year after child birth. J Fam Reprod Health. (2016) 10:146–53. PMC5241359 28101116

[28] Giallo R Rose N Vittorino R. Fatigue, wellbeing and parenting in mothers of infants and toddlers with sleep problems. J Reprod Infant Psychol. (2011) 29:236–49. 10.1080/02646838.2011.593030

[29] Sanders R Lehmann J Gardner F. Parents’ experiences of early parenthood – preliminary findings. Child Aust. (2014) 39:185–94. 10.1017/cha.2014.20

[30] Katz LF Maliken AC Stettler NM. Parental meta-emotion philosophy: a review of research and theoretical framework. Child Dev Perspect. (2012) 6:417–22. 10.1111/j.1750-8606.2012.00244.x

[31] Meyer S Raikes HA Virmani EA Waters S Thompson RA. Parent emotion representations and the socialization of emotion regulation in the family. Int J Behav Dev. (2014) 38:164–73. 10.1177/0165025413519014

[32] Morris AS Criss MM Silk JS Houltberg BJ. The impact of parenting on emotion regulation during childhood and adolescence. Child Dev Perspect. (2017) 11:233–8. 10.1111/cdep.12238

[33] Rutherford HJV Wallace NS Laurent HK Mayes LC. Emotion regulation in parenthood. Dev Rev. (2015) 36:1–14. 10.1016/j.dr.2014.12.008 26085709 PMC4465117

[34] Cabrera NJ Tamis-LeMonda CS. Handbook of Father Involvement: Multidisciplinary Perspectives. New York: Routledge (2002).

[35] Lamb ME. The Role of the Father in Child Development. Hoboken, NJ: John Wiley & Sons (2010).

[36] Devi PR Priya M. Father’s involvement in child care and development: a pilot study from Coimbatore. Int J Home Sci. (2019) 5:414–6.

[37] Goel Y Mishra S. Cultural understandings of fathering and fatherhood in India: an exploration of lived experiences. Cult Psychol. (2024) 30:245–78. 10.1177/1354067X231154006

[38] Frankel LA Umemura T Jacobvitz D Hazen N. Marital conflict and parental responses to infant negative emotions: relations with toddler emotional regulation. Infant Behav Dev. (2015) 40:73–83. 10.1016/j.infbeh.2015.03.004 26047678

[39] Holland AS McElwain NL. Maternal and paternal perceptions of coparenting as a link between marital quality and the parent–toddler relationship. J Fam Psychol. (2013) 27:117–26. 10.1037/a0031427 23421839

[40] Sudo M Low PHX Kyeong Y Meaney MJ Kee MZL Chen H Grandparents’ and domestic helpers’ childcare support: implications for well-being in Asian families. J Marriage Fam. (2025) 87:134–56. 10.1111/jomf.13010

[41] Ghorbani S Gharraee B Hosseini F Maghami Sharif Z Aghebati A. Changing parenting style between two generations and its impacts on the severity of behavioral and emotional symptoms. Asia-Pac Psychiatry. (2022) 14:e12448. 10.1111/appy.12448 33480179

[42] Karmakar R. The impact of perception of consistency and inconsistency in parenting style on pro-social motives of adolescents. Soc Psychol Soc. (2017) 8:101–15. 10.17759/sps.2017080207

[43] Sahithya BR Manohari SM Vijaya R. Parenting styles and its impact on children – a cross cultural review with a focus on India. Ment Health Relig Cult. (2019) 22:357–83. 10.1080/13674676.2019.1594178

[44] Tan H Yow S Asano C. Feeding-related knowledge, attitudes, and practices among grandparents in Singapore. Nutrients. (2019) 11:1696. 10.3390/nu11071696 31340578 PMC6683024

[45] Sanders M. Triple P-positive parenting program as a public health approach to strengthening parenting. J Fam Psychol. (2008) 43(22):506–17. 10.1037/0893-3200.22.3.506 18729665

[46] Umemura T Jacobvitz D Messina S Hazen N. Do toddlers prefer the primary caregiver or the parent with whom they feel more secure? The role of toddler emotion. Infant Behav Dev. (2013) 36:102–14. 10.1016/j.infbeh.2012.10.003 23268105

[47] Creswell JW. A Concise Introduction to Mixed Methods Research. Thousand Oaks, CA: SAGE Publications (2015).

