# Supplementary material for: Personal and familial needs of parents with toddlers: a qualitative study exploring the needs of parents in urban Bengaluru, India
Source: Front Child Adolesc Psychiatry. 2026 May 25;5:1664652. doi: 10.3389/frcha.2026.1664652 (PMC13243268; doi:10.3389/frcha.2026.1664652)
Supplement: Supplementary file 1 [file Table1.docx]

Supplementary Material

**Semi-structured Interview Schedule - Pr**

This Semi-structured Interview Schedule has been prepared to use with professionals working closely with the parents of toddlers, such as Psychiatrists, Psychologists, Social workers, ICDS officers, and Paediatricians.

Aim: To explore the experiences of the professionals while working with the parents of toddlers and their concerns related to parenting.

Name: Age:

Qualification: Gender:

Designation:

Years of experience in the field:

1. What are your observations while working with the parents of toddlers?

Specific prompts

- 1. Nature of problems the parents come with
  2. Strengths in the parents/ resources in the family
  3. Expectations of parents
  4. General doubts parents ask you about the toddlers
  5. Risk factors associated with parenting
  6. Ways of early identification

1. What are the needs of the parents while parenting the toddlers?

Specific prompts

- 1. Toddler related specific issues affecting parenting practices
  2. Parenting related aspects that affect toddlers
     1. Common discipline strategies
     2. Parent child relationship
     3. Toddlers experience neglect/ no supervision?
  3. Role of culture in parenting
     1. Shared caregiving
     2. Differences in caregiving
     3. Religious/ faith healing and its influences
  4. Any barriers to change parenting style
  5. Any other factors affecting parenting
     1. Role of sibling
     2. Parents’ early childhood experience of parenting

1. What do you think are the areas the parents have to improve with respect to the parenting practice?

Specific prompts

- 1. Knowledge regarding parenting
  2. Parenting Practices
  3. Attitudes
  4. Consistency between parents
  5. Personal self-care
  6. Stress management
  7. Any other areas?

1. Any other suggestions

**Semi-structured Interview Schedule – Pa**

1. Could you talk about your understanding of child development and parenting?
   1. The areas of child development
   2. Source of knowledge regarding parenting skills
   3. Can you describe what parenting skills are?
   4. What do you think about the role of gender of the child in his/her development?
2. Can you talk about your experience of nurturing your child?
   1. How do you feel about handling the child?
   2. Could you list out the behaviours of the child you find difficult to manage?
   3. How do you respond to them?
   4. What is your child good at?
   5. Please share your challenges related to parenting your child.
3. Who are all involved in the caretaking of the child? How is it done?
   1. How much time do you spend with your child?
   2. How do you spend time with your child?
   3. How do you play with the child?
   4. Who do you think should give more attention and time to the child?
   5. What are your family and your strengths related to childcare?
4. How do you come to a consensus with your partner over parenting decisions?
   1. How does your partner respond to your parenting?
   2. How do you respond to your partner’s parenting?
   3. How is your communication with your partner?
   4. How do you resolve disagreements or arguments?
5. How did you learn about parenting? Could you tell me more about the sources of your knowledge?
   1. Could you share your childhood experience of parenting?
6. How do you feel about the way you are handling the child?
7. Do you have any queries related to the child’s behavioural and emotional development?
   1. Specific to your child
   2. Child in general

**Socio-demographic details of parents collected at Phase II**

**Table 1 - Socio-demographic details – Age & income**

| **Variable** | **Mean (SD)** | **Minimum – maximum** | **Q_2_ (Q_1_, Q_3_)** |
| --- | --- | --- | --- |
| Child Age (in months) | 27.2 (7.1) | 12 - 35 | 29 (23.2, 33.0) |
| Mother age (in years) | 30.5 (4.2) | 21 – 39 | 31 (27.0, 34.0) |
| Father age (in years) | 35.4 (4.0) | 29 – 46 | 35 (32.0, 38.0) |
| Monthly family income (₹) | 142291.6 (71681.6) | 25000 - 300000 | 150000.0  (82500.0, 200000.0) |

Q_1_=25th percentile; Q_2_=50th percentile; Q_3_=75th percentile

**Table 2 – Frequency distribution of socio-demographic details of the toddlers and parents**

| **Variable** | | | **n** | **%** |
| --- | --- | --- | --- | --- |
| Parents participated in the study | | Only fathers | 1 | 2.1 |
|  |  | Only mothers | 7 | 14.6 |
|  |  | Both parents | 40 | 83.3 |
| Gender of the child | | Male | 21 | 43.8 |
|  |  | Female | 27 | 56.3 |
| Number of siblings | | No sibling | 34 | 70.8 |
|  |  | One | 13 | 27.1 |
|  |  | Two | 1 | 2.1 |
| Birth order of index child | | First | 35 | 72.9 |
|  |  | Second | 12 | 25.0 |
|  |  | Third | 1 | 2.1 |
| Education qualification | Father  (N=41) | ≤ Graduation | 6 | 14.6 |
|  |  | Graduation | 22 | 53.7 |
|  |  | Post-graduation | 13 | 31.7 |
|  | Mother (N=47) | ≤ Graduation | 4 | 8.5 |
|  |  | Graduation | 27 | 57.4 |
|  |  | Post-graduation | 16 | 34.0 |
| Current working status – Employed | | Father | 41 | 100.0 |
|  |  | Mother | 23 | 48.9 |
| Religion | | Hindu | 40 | 83.3 |
|  |  | Muslim | 8 | 16.7 |
| Type of family | | Nuclear | 24 | 50.0 |
|  |  | Joint/ extended | 24 | 50.0 |
